# Insomnia nosology: a systematic review and critical appraisal of historical diagnostic categories and current phenotypes

**DOI:** 10.1111/jsr.13910

**Published:** 2023-04-30

**Authors:** Casandra C. Nyhuis, Julio Fernandez-Mendoza

**Affiliations:** 1 Department of Public Health Sciences, Pennsylvania State University College of Medicine, Hershey, Pennsylvania, USA; 2 Sleep Research and Treatment Center, Department of Psychiatry and Behavioral Health, Pennsylvania State University College of Medicine, Hershey, Pennsylvania, USA

**Keywords:** clusters, insomnia, nosology, phenotyping, subtyping

## Abstract

Insomnia nosology has significantly evolved since the Diagnostic and Statistical Manual (DSM)-III-R first distinguished between ‘primary’ and ‘secondary’ insomnia. Prior International Classification of Sleep Disorders (ICSD) nosology ‘split’ diagnostic phenotypes to address insomnia’s heterogeneity and the DSM nosology ‘lumped’ them into primary insomnia, while both systems assumed causality for insomnia secondary to health conditions. In this systematic review, we discuss the historical phenotypes in prior insomnia nosology, present findings for currently proposed insomnia phenotypes based on more robust approaches, and critically appraise the most relevant ones. Electronic databases PsychINFO, PubMED, Web of Science, and references of eligible articles, were accessed to find diagnostic manuals, literature on insomnia phenotypes, including systematic reviews or meta-analysis, and assessments of the reliability or validity of insomnia diagnoses, identifying 184 articles. The data show that previous insomnia diagnoses lacked reliability and validity, leading current DSM-5-TR and ICSD-3 nosology to ‘lump’ phenotypes into a single diagnosis comorbid with health conditions. However, at least two new, robust insomnia phenotyping approaches were identified. One approach is multidimensional-multimethod and provides evidence for self-reported insomnia with objective short versus normal sleep duration linked to clinically relevant outcomes, while the other is multidimensional and provides evidence for two to five clusters (phenotypes) based on self-reported trait, state, and/or life-history data. Some approaches still need replication to better support whether their findings identify true phenotypes or simply different patterns of symptomatology. Regardless, these phenotyping efforts aim at improving insomnia nosology both as a classification system and as a mechanism to guide treatment.

## 1 | INTRODUCTION

Diagnosing insomnia was historically a challenge due to the lack of precise and universally accepted diagnostic criteria. While the scientific community evolved from considering insomnia a symptom (National Institute of Mental Health [NIMH], 1984) to an independent sleep disorder in its own right (National Institutes of Health [NIH], 2005), there has existed high variability in how insomnia disorder has been defined (Edinger et al., 2004). Insomnia is classified and diagnosed based on the criteria outlined by various diagnostic systems, including the Diagnostic and Statistical Manual (DSM), the International Classification of Sleep Disorders (ICSD), and the International Classification of Diseases (ICD). Insomnia nosology has evolved since its addition to the DSM-III-R (American Psychiatric Association [APA], 1987), where ‘general insomnia diagnostic criteria’ were first proposed (Table 1) and a distinction was made between ‘primary’ and ‘secondary’ insomnia depending on whether the purported aetiology of insomnia was causally attributed to underlying medical or psychiatric conditions. Currently, a single insomnia disorder diagnosis is considered in the ‘lumping’ approach of the DSM-5-TR (APA, 2022), ICSD-3 (American Academy of Sleep Medicine [AASM], 2014), and ICD-11 (World Health Organization [WHO], 2019). Regardless of the current nosology used, an insomnia disorder diagnosis is made based on the individual’s self-reported night-time sleep and daytime functioning symptoms, for which chronicity can be specified, and where no causal attributions are made as it pertains to the presence of physical or mental health comorbidities.

Despite the current ‘*one ring to rule them all*’ nosology, it has been long recognised that insomnia is a heterogeneous disorder. Over the past 40 years, there have been several delineated diagnostic phenotypes that failed to demonstrate optimal reliability and validity (Edinger et al., 2011). More recently, efforts have been made to identify insomnia phenotypes associated with clinically relevant outcomes via objectively measured sleep dimensions different than insomnia symptoms per se or via cognitive-emotional dimensions and life history. In this systematic review, we will first cover the historical phenotypes in prior insomnia nosology that led to the present-day definition of insomnia disorder. Second, we will present findings for the currently proposed insomnia phenotypes. Finally, we will critically appraise these insomnia phenotypes based on their supportive literature.

## 2 | METHODS

The search strategy and selection criteria of this systematic review was carried out in accordance with the Preferred Reporting Items for Systematic Reviews and Meta-Analyses (PRISMA) guidelines (Page et al., 2021). PsychINFO, PubMED, and Web of Science were searched using a combination of keyword and Medical Subject Headings (MeSH) terms. Keywords for all fields combined the following terms: (1) ‘insomnia’ or ‘insomnia disorder’, or ‘primary insomnia’ or ‘insomnia syndrome’ or ‘chronic insomnia’ or ‘insomnia symptoms’ and (2) ‘type’ or ‘subtyp*’ or ‘phenotyp*’. MeSH terms were added to the keywords to refine search results and used on their own to capture papers that were missed through the keyword search. No limits or filters were placed on search results. The results captured papers published in English between 1976 and 2022 (the year this review was conducted). The initial search was originally conducted July 2022 and was repeated in December 2022 to capture new literature. Articles and handbooks were included upon agreement by both authors after a review of the abstracts if they: were diagnostic manuals, attempted to identify phenotypes of insomnia, conducted their own systematic review or meta-analysis of insomnia phenotyping, and/or assessed the reliability or validity of insomnia phenotype diagnostic criteria. Both authors also examined the references of eligible articles to identify articles missed through literature searches.

This search strategy enabled us to capture literature that not only assessed and discussed phenotypes outlined in diagnostic systems, but to also include the work performed in the field that leverages multiple approaches to identify new potential phenotypes and their potential clinical and health outcomes. As a result, our search also captured publications that examined the association between insomnia and various clinical and health outcomes, which are discussed herein only if pertinent to insomnia phenotyping efforts.

## 3 | RESULTS

**FIGURE 1:**
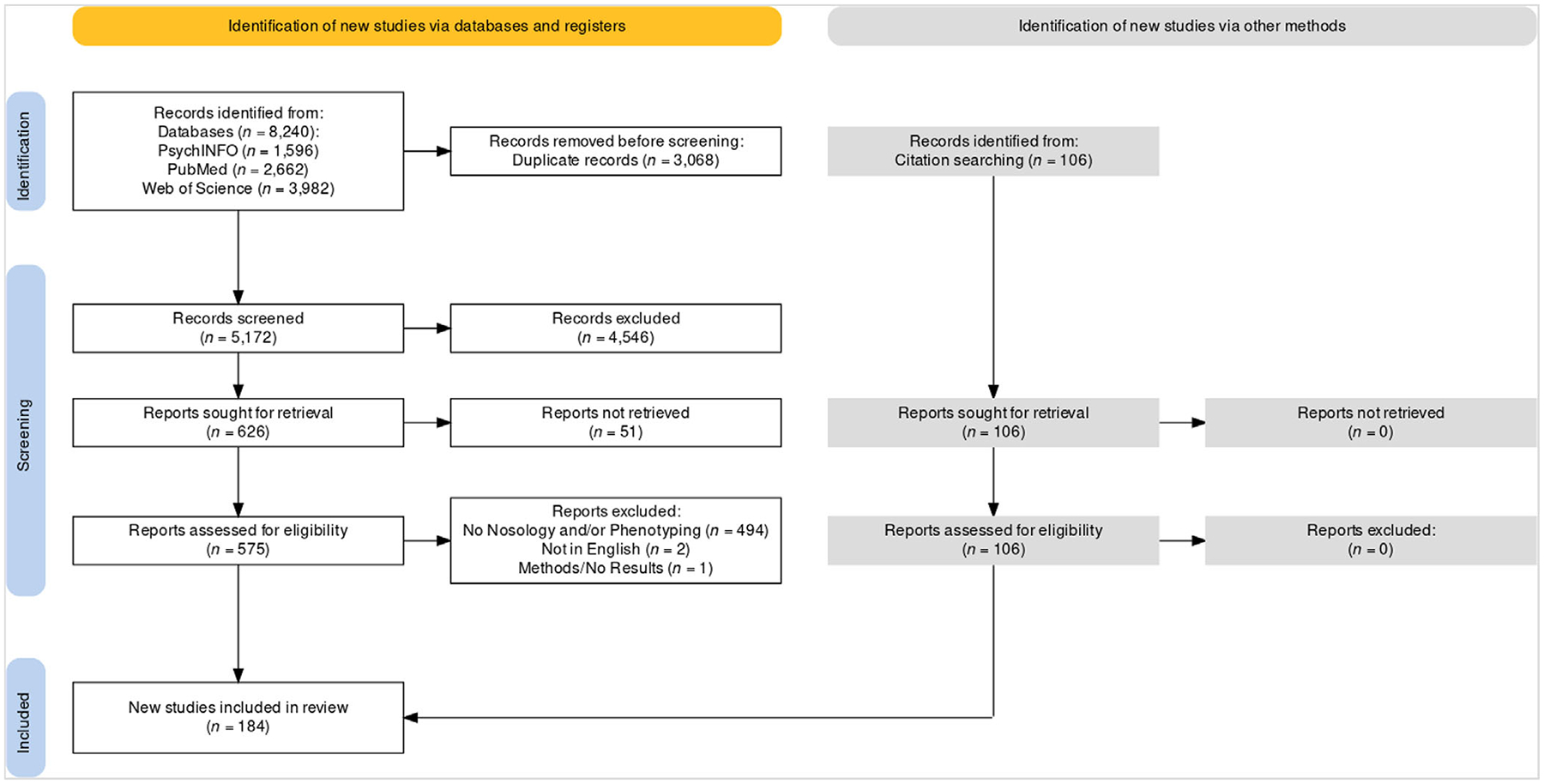
Preferred Reporting Items for Systematic Reviews and Meta-Analyses (PRISMA) flow diagram of the studies obtained through a literature search and screened for inclusion in the present study. The flow diagram was created using the R Package and Shiny App from Haddaway et al. (2022).

The search yielded 8240 articles that were assessed for inclusion (Figure 1); of those 3068 were duplicates and an additional 4546 were screened and excluded. In all, 626 articles were sought for additional screening but only 575 of those articles could be found. Of these 575, 494 did not address insomnia nosology or phenotyping, two were in a language neither author could read, and one article was relevant to the topic but was a methods paper and contained no results. This yielded a total of 78 articles from the literature search. An additional 106 articles were identified from the references of the 78 eligible articles, resulting in a total of 184 articles that will be discussed in this systematic review.

### 3.1 | Insomnia diagnoses and nosology phenotypes

The DSM-III-R (APA, 1987) and the ICSD (American Sleep Disorders Association [ASDA], 1990) provided initial criteria for diagnosing insomnia using very different approaches. The DSM-III-R categorised insomnia disorder using a ‘lumping’ approach where general insomnia diagnostic criteria were provided and only a singular diagnosis not secondary to a known psychiatric or medical disorder was allowed: primary insomnia (Table 2). In contrast, both the ICSD and ICSD-R (AASM, 1997) ‘split’ insomnia into various diagnostic phenotypes to account for the purported underlying aetiology, pathophysiology, natural course, and clinical characteristics. The ‘primary’ insomnia phenotypes in the ICSD-R for adults included: psychophysiological insomnia (previously referred in research studies as ‘objective insomnia’ [Dorsey & Bootzin, 1997; Sugerman et al., 1985]), sleep state misperception (previously referred to as ‘subjective insomnia’ [Dorsey & Bootzin, 1997; Sugerman et al., 1985]), idiopathic insomnia, and adjustment insomnia sleep disorder (Table 2). In preparation for the DSM-IV, a scientific expert workgroup reviewed the literature regarding these ICSD phenotypes and tested them in field studies to determine if they should be included within the DSM-IV and subsequent DSM-IV-TR (APA, 2000). These experts did not find sufficient evidence to abandon the DSM’s ‘lumping’ approach given the low reliability and diagnostic agreement in the field study for ICSD’s insomnia phenotypes (Reynolds et al., 1991).

With these issues in mind, the AASM commissioned a workgroup to derive a standardised set of research diagnostic criteria (RDC) consisting of a valid and reasonable list of insomnia phenotypes after a review of the literature (Edinger et al., 2004). Similar to the historical DSM-III-R, Edinger et al. (2004) proposed a set of general diagnostic criteria for insomnia disorder as a starting point for improving insomnia research and push the field forward (Table 1). In addition, the main adult phenotypes in the RDC that were also not secondary to other known disorders were reduced to: primary insomnia, psychophysiological insomnia, paradoxical insomnia, and idiopathic insomnia. This RDC effort informed the ICSD-2 and, consequently, the main insomnia phenotypes that were not secondary to other known disorders in adults included: psychophysiological insomnia, paradoxical insomnia, idiopathic insomnia, and adjustment insomnia/acute insomnia (Table 2).

Psychophysiological insomnia, as described in the ICSD-R (AASM, 1997), was characterised by the individual having a state of heightened arousal, learned sleep preventing associations, and decreased functioning during wakefulness. In addition to a subjective complaint of conditioned arousal (e.g., inability to fall asleep at bedtime but falling asleep easily during monotonous activities) or somatised tension (e.g., muscle tension in bed), polysomnography (PSG) monitoring had to demonstrate either an ‘increased sleep latency’, ‘reduced sleep efficiency’, and/or an ‘increased number and duration of awakenings’ that could not be explained by other medical, mental, or sleep disorders. The ICSD-R did not specify quantitative thresholds for any of that PSG criteria. Furthermore, both the RDC and ICSD-2 explicitly removed the criterion pertaining to PSG-verified sleep disturbance to rely solely on self-reported data for a diagnosis of psychophysiological insomnia to be made, emphasising patient-reported sleep-related anxiety/preoccupation, cognitive arousal, and conditioned somatic tension in bed as essential features of the phenotype.

Paradoxical insomnia (also known as sleep state misperception in the ICSD and ICSD-R), was characterised in the ICSD-2 by a chronic pattern of little or no sleep most nights, sleep log data that indicated insufficient sleep compared to published age-adjusted normative values, no indication of sleep for several nights per week, an absence of daytime naps following such nights, and a constant mismatch or discrepancy between objective findings from PSG or actigraphy (ACT) and subjective sleep estimates, referred to as ‘sleep misperception’ (Harvey & Tang, 2012; Te Lindert et al., 2020). Additionally, individuals would typically report near constant awareness of environmental stimuli throughout most nights and/or a pattern of conscious thoughts throughout most nights while in bed, as well as daytime impairments that were much less severe than expected, given the extreme level of self-reported sleep deprivation (AASM, 2005). With these diagnostic criteria, paradoxical insomnia was considered to affect only 5% of all individuals with insomnia (AASM, 2005; Edinger & Krystal, 2003).

A total of 13 studies out of 17 showed that psychophysiological insomnia or ‘objective insomnia’ was associated with impairment in objectively measured neurocognitive performance (Backhaus et al., 2006; Bastien et al., 2003; Bonnet & Arand, 1995; Edinger et al., 2000b; Edinger et al., 2008; Fang et al., 2008; Haimov et al., 2008; Hauri, 1997; Nissen et al., 2011; Orff et al., 2007; Seidel et al., 1984; Szelenberger & Niemcewicz, 2000; Varkevisser & Kerkhof, 2005), while five studies out of 17 did not show paradoxical insomnia or ‘subjective insomnia’ to be associated with objectively measured neurocognitive performance (Bonnet & Arand, 1997; Broman et al., 1992; Lundh et al., 1997; Orff et al., 2007; Varkevisser et al., 2007).

As shown in in Table 3, 17 studies examined the sleep microarchitecture of ICSD-2 psychophysiological insomnia and paradoxical insomnia using quantitative electroencephalography (qEEG) techniques such as:

- *Cortical arousability and its asymmetry* (Bastien et al., 2008; Bastien et al., 2013; Ceklic & Bastien, 2015; Chouvarda et al., 2013; Parrino et al., 2009; Pérusse et al., 2013; Rodenbeck et al., 2000; St-Jean et al., 2012; St-Jean et al., 2013; Turcotte et al., 2011; Turcotte & Bastien, 2009).
- *K-complexes* (Bastien et al., 2009b; Bastien et al., 2009a; Forget et al., 2011).
- *Sleep spindles* (Normand et al., 2016).
- *Wake intrusions in rapid eye movement (REM) sleep and dream content* (Feige et al., 2008; Feige et al., 2018; Feige et al., 2021; Pérusse et al., 2015; Pérusse et al., 2016).

Based on these studies, Bastien et al. (2013) and others hypothesised that psychophysiological insomnia may be a disorder of inability to inhibit information processing during sleep onset and non-REM (NREM) sleep, while paradoxical insomnia may be a disorder of overall enhanced attentional processing resulting in inhibition deficits during sleep onset, NREM and REM sleep (Riemann et al., 2012; Turcotte et al., 2011).

However, a large study investigated the reliability and validity of the insomnia diagnostic phenotypes in the DSM-IV-TR and ICSD-2 reviewed above (Edinger et al., 2011). Edinger et al. (2011) used three pairs of clinicians at two sites who used one of three different interview approaches and found that only insomnia related/due to a mental disorder and insomnia due to a medical condition from either nosology were considered ‘highly acceptable’ and ‘acceptable’, respectively. Insomnia due to an alcohol-related sleep disorder from the DSM-IV-TR and idiopathic insomnia from the ICSD-2 were considered ‘marginally acceptable’. In contrast, primary insomnia from the DSM-IV-TR and psychophysiological insomnia and paradoxical insomnia from the ICSD-2 were considered ‘unacceptable’. Consistently, systematic review has shown moderate-to-low agreement (mean Cohen’s kappa = 0.61) between the patient-reported outcomes used to assess insomnia and the disorder’s diagnostic criteria in prior nosology (Gauld et al., 2022).

### 3.2 | Objective measures in insomnia phenotyping

As reviewed above, PSG was part of the diagnostic criteria for psychophysiological and idiopathic insomnia in the ICSD and ICSD-R, albeit without specifying thresholds for PSG parameters, as well as for paradoxical insomnia in the ICSD, ICSD-R, RDC and ICSD-2, where there was no clear consensus or formal thresholds either for a precise definition (Castelnovo et al., 2019). The present subsection will review phenotyping approaches using objective measures differently than prior insomnia nosology.

#### 3.2.1 | Night-time sleep duration

**FIGURE 2:**
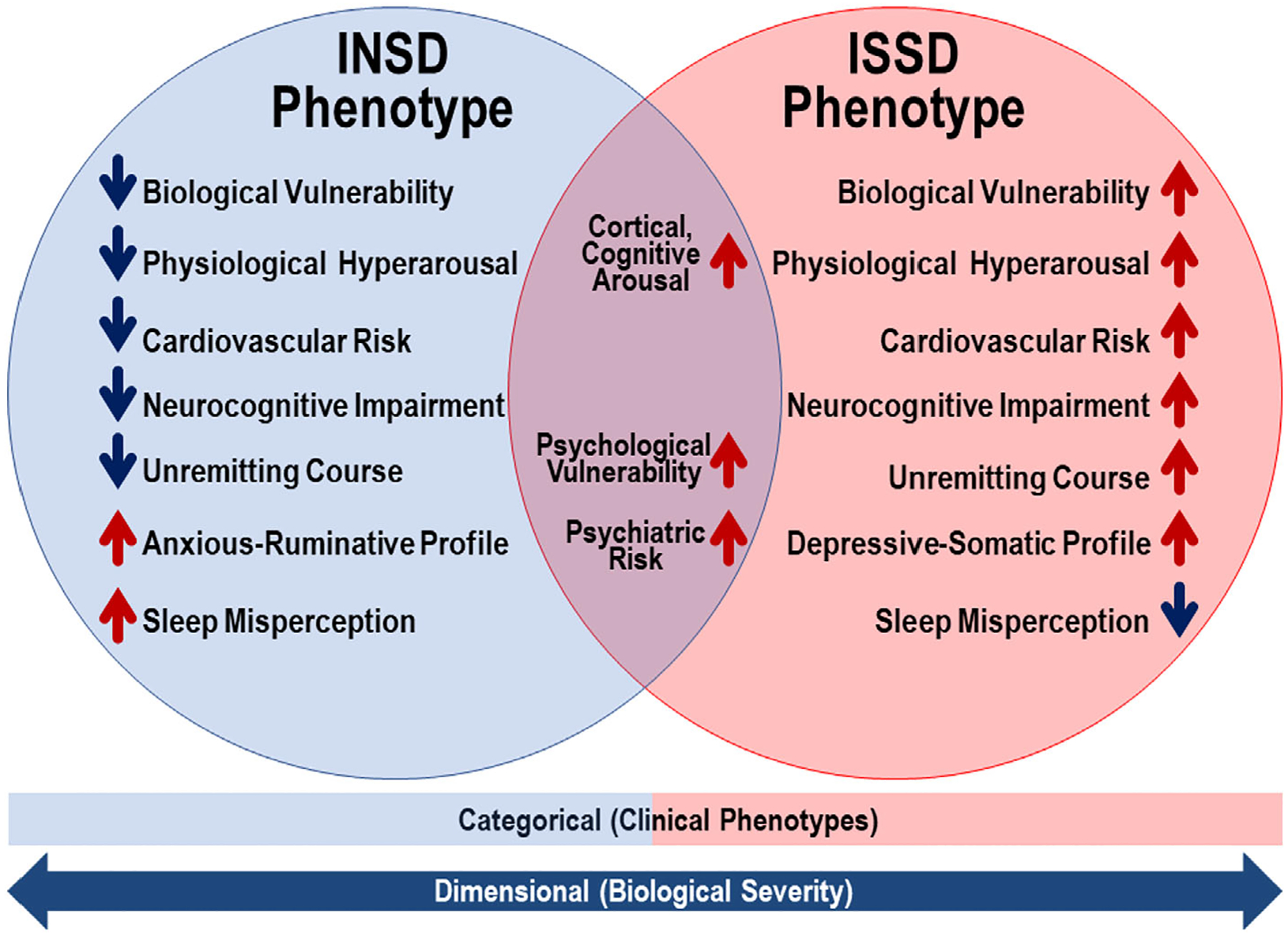
Insomnia phenotypes based on objective sleep duration. The insomnia with short sleep duration (ISSD) phenotype is associated with physiological hyperarousal, as index by a dysregulated hypothalamic–pituitary–adrenal axis (e.g., cortisol levels), sympathetic–adrenal medullary axis (e.g., noradrenaline levels), and cardiac autonomic modulation (e.g., blunted heart rate variability), with increased cardiometabolic risk (e.g., hypertension, diabetes, heart disease) and neurocognitive impairment (e.g., executive deficits); its aetiology is purported to carry greater biological vulnerability (e.g., genetics, stress-system reactivity). Individuals with the ISSD phenotype have also been shown to have a persistent, unremitting natural course, to present with a profile characterised by somatic complaints and depressed mood, and to accurately estimate, or even overestimate, their sleep duration. In contrast, insomnia with normal sleep duration (INSD) phenotype is not associated with physiological hyperarousal, cardiometabolic risk, or neurocognitive impairment. Individuals with the INSD phenotype have been shown to have higher remission rates in their natural course, to present with a profile characterised by anxious-ruminative traits, greater dysfunctional sleep-related beliefs, poor coping resources, and to underestimate their sleep duration (i.e., ‘sleep misperception’). Both phenotypes have been shown to present with cognitive and cortical arousal, as indexed by electroencephalography measures of increased information processing during the pre-sleep period and while asleep, greater psychological vulnerability (i.e., emotional reactivity), and increased psychiatric risk, albeit through different psychobiological mechanisms (e.g., poor coping resources in the INSD phenotype). In addition, preliminary meta-analytical data suggest a two-times higher full remission rate in the INSD phenotype than the ISSD phenotype after cognitive-behavioural treatment of insomnia. Taken with permission from Fernandez-Mendoza J. *Insomnia with Objective Short Sleep Duration*. In: Kushida CA, Ed, Encyclopedia of Sleep and Circadian Rhythms, Reference Module in Neuroscience and Biobehavioral Psychology, 2nd Edition. Elsevier Academic Press, New York, February 2021, 1–9. doi.org/10.1016/B978-0-12-822963-7.00013-X.

Over the last 20 years, there has been a renewed interest in using multidimensional-multimethod approaches to sleep health, including the use of objective measures of total sleep time (TST) to phenotype insomnia. In the late 1990s, Vgontzas and colleagues showed that the degree of PSG-measured total wake time, wake after sleep onset (WASO) and other markers of sleep disturbance across four consecuitve nights were associated with hyperactivity of the hypothalamic–pituitary–adrenal (HPA) and sympathetic–adrenal medullary (SAM) axes (Vgontzas et al., 1998) in individuals with insomnia or primarily found in those with insomnia and short objective TST (Vgontzas et al., 2001). Based on those early findings, Vgontzas and colleagues examined in 2009 the joint association between self-reported insomnia and objective short sleep duration on clinically relevant outcomes such as hypertension (Vgontzas et al., 2009a) and diabetes (Vgontzas et al., 2009b). These, and subsequent data reviewed below, led investigators to propose the insomnia with short sleep duration (ISSD) and insomnia with normal sleep duration (INSD) phenotypes shown in Figure 2, which differ in pathophysiology, health outcomes, clinical features, natural course, and treatment response (Fernandez-Mendoza, 2017; Vgontzas et al., 2013).

The ISSD phenotype, defined in adults as self-reporting insomnia disorder and sleeping objectively for <6 h (Vgontzas et al., 2013), has been characterised by 24-h physiological hyperarousal in 21 studies as measured by:

- *Increased alertness/decreased physiological sleep propensity* (Bonnet & Arand, 1995; Bonnet & Arand, 1998b; Dorsey & Bootzin, 1997; Sugerman et al., 1985).
- *Sympathetic/parasympathetic imbalance* (Bonnet et al., 2014; Bonnet & Arand, 1995; Bonnet & Arand, 1998a; Huang et al., 2018; Jarrin et al., 2018b; Miller et al., 2016; Spiegelhalder et al., 2011).
- *Stress system (HPA and SAM axes) activation* (Castro-Diehl et al., 2015; Castro-Diehl et al., 2016; D’Aurea et al., 2015; Vgontzas et al., 2001).
- *Immune system activation* (Lerman et al., 2022; Tempaku et al., 2018; Vgontzas et al., 2002).
- *Central nervous system (CNS) activation* (Fan et al., 2019; Krystal et al., 2002; Miller et al., 2017; Spiegelhalder et al., 2012).

The pathophysiological findings above include prior studies using PSG sleep efficiency of <85% or similar objective metrics to define ISSD and have been supported also by correlational studies where the degree of PSG-measured sleep disturbance was associated with higher activity of the HPA and SAM axes (Floam et al., 2015; Rodenbeck et al., 2002; Vgontzas et al., 1998), greater alertness/lower physiological sleep propensity (Bonnet & Arand, 2010; Roehrs et al., 2011; Stepanski et al., 1988) or increased CNS network activation (Regen et al., 2016; Spiegelhalder et al., 2012; Spiegelhalder et al., 2016) in individuals with insomnia.

As shown in Figure 2, the ISSD phenotype has also been associated with specific adverse health outcomes in 37 studies:

- *Cardiovascular* (Bathgate et al., 2016; Bertisch et al., 2018; Fernandez-Mendoza et al., 2012; Fernandez-Mendoza et al., 2018; Hein et al., 2019a; Hein et al., 2019b; Huang et al., 2018; Nakazaki et al., 2012; Sigurdardottir et al., 2022; Vgontzas et al., 2009a).
- *Metabolic* (Castro-Diehl et al., 2018; D’Aurea et al., 2015; Duan et al., 2023; Hein et al., 2018; Miner et al., 2022; Vasisht et al., 2013; Vgontzas et al., 2009b).
- *Neurocognitive* (Biddle et al., 2017; Bonnet & Arand, 1995; Bonnet & Arand, 1997; Fan et al., 2019; Fernandez-Mendoza et al., 2010; Fernandez-Mendoza et al., 2021; Khassawneh et al., 2018; Miller et al., 2016; Miller et al., 2017; Olaithe et al., 2021).
- *Psychiatric* (Biddle et al., 2019; Bonnet & Arand, 1997; Dorsey & Bootzin, 1997; Edinger et al., 2000a; Fernandez-Mendoza et al., 2011; Fernandez-Mendoza et al., 2015; Miner et al., 2022; Saulnier et al., 2022; Vgontzas et al., 2012).
- *Frailty or worse prognosis of comorbid health conditions* (Basta et al., 2022; Lerman et al., 2022; Miner et al., 2022).
- *Mortality in men* (Vgontzas et al., 2010).

Although Johann et al. (2017) reported that the ISSD phenotype (*n* = 64) was not associated with significantly increased odds of hypertension (odds ratio [OR] = 1.82) or diabetes (OR = 2.30) when compared to those with INSD (*n* = 264), a meta-analysis of 10 studies (Bathgate et al., 2016; Bertisch et al., 2018; Crönlein et al., 2020; Fan et al., 2019; Hein et al., 2019b; Jarrin et al., 2018a; Jarrin et al., 2018b; Johann et al., 2017; Miller et al., 2016; Vasisht et al., 2013; Vgontzas et al., 2010) showed a significantly higher pooled-risk of hypertension (relative risk [RR] = 1.54) and type 2 diabetes (RR = 1.63) in the ISSD phenotype (*n* = 532) when compared to the INSD phenotype (*n* = 468; Johnson et al., 2021). Of note, Bertisch et al. (2018), despite reporting a 30% significantly higher risk of incident cardiovascular disease in the ISSD phenotype (*n* = 354) compared to good sleepers (*n* = 350), could not replicate the association with mortality (hazard ratio = 1.07) reported by Vgontzas et al. (2010).

From a clinical features standpoint, the ISSD phenotype has been characterised by accurately estimating, or even overestimating, the objective TST they have been defined upon in six studies (Bathgate et al., 2016; Bathgate et al., 2017; Bertisch et al., 2018; Fernandez-Mendoza et al., 2011; Jarrin et al., 2018b; Lovato et al., 2021), with only 10% of them having ‘sleep misperception’ (i.e., underestimating their objective TST by ≥1 h) (Fernandez-Mendoza et al., 2011). The ISSD has also been characterised by depressive-somatic traits in two studies (Edinger et al., 2000a; Fernandez-Mendoza et al., 2011) and a persistent natural course or longer-lasting duration in three studies (Fernandez-Mendoza et al., 2012; Johann et al., 2017; Vgontzas et al., 2012).

From a developmental standpoint, the ISSD phenotype, defined in youth as having parent- or self-reported insomnia and sleeping objectively for <7.5 h (Fernandez-Mendoza, 2017), has been associated in six studies with increased cortisol levels (Fernandez-Mendoza et al., 2014), increased beta EEG power during sleep onset latency (SOL) and NREM sleep (Fernandez-Mendoza et al., 2016a), increased C-reactive protein levels (Fernandez-Mendoza et al., 2017), clinically elevated internalising symptoms (Calhoun et al., 2017; Fernandez-Mendoza et al., 2016b), and greater likelihood of worsening into adult insomnia disorder (Fernandez-Mendoza et al., 2022).

From a therapeutic standpoint, the ISSD phenotype has been associated with a poor response to cognitive-behavioural treatment of insomnia (CBT-I) in adults. A meta-analysis (He et al., 2022) of seven studies (Bathgate et al., 2017; Crönlein et al., 2020; Kalmbach et al., 2020; Lovato et al., 2016; Miller et al., 2018; Rochefort et al., 2019; Troxel et al., 2013) that performed retrospective secondary analyses of prior randomised clinical trials (RCTs) by splitting their original sample into ISSD and INSD at pre-treatment, found a low 32% (95% confidence interval [CI] 17%–46%) remission rate in the ISSD phenotype. In addition, a small pilot study showed trazodone to decrease cortisol levels and lengthen ACT-measured TST in the ISSD phenotype (Vgontzas et al., 2020).

As shown in Figure 2, the INSD phenotype, defined in adults as self-reporting insomnia disorder and sleeping objectively for >6 h (Vgontzas et al., 2013), is characterised by normal activity of the HPA and SAM axes and normal levels of alertness/physiological sleep propensity (Bonnet & Arand, 1997; Castro-Diehl et al., 2015; Castro-Diehl et al., 2016; Dorsey & Bootzin, 1997; Fan et al., 2019; Miller et al., 2016; Miller et al., 2017; Spiegelhalder et al., 2011; Sugerman et al., 1985; Tempaku et al., 2018; Vgontzas et al., 2001) but increased cortical arousal during NREM sleep (Krystal et al., 2002; Spiegelhalder et al., 2012).

The INSD phenotype has been associated with increased risk of psychiatric morbidity, such as incident depression and suicidal ideation and attempts (Fernandez-Mendoza et al., 2015; Saulnier et al., 2022), but not with cardiovascular (Bertisch et al., 2018; Fernandez-Mendoza et al., 2012; Fernandez-Mendoza et al., 2018; Vgontzas et al., 2009a), metabolic (Castro-Diehl et al., 2018; Hein et al., 2018; Vgontzas et al., 2009b) or neurocognitive (Fan et al., 2019; Fernandez-Mendoza et al., 2010; Fernandez-Mendoza et al., 2021; Khassawneh et al., 2018) morbidity or mortality (Bertisch et al., 2018; Vgontzas et al., 2010).

As it pertains to clinical features, the INSD phenotype has been characterised by underestimating the objective TSTs they have been defined upon (Bathgate et al., 2016; Bathgate et al., 2017; Fernandez-Mendoza et al., 2011; Jarrin et al., 2018b; Lovato et al., 2021), with 55% of them having ‘sleep misperception’ as defined above (Fernandez-Mendoza et al., 2011). The INSD has also been characterised by elevated scores in state- or trait-scales (e.g. Minnesota Multiphasic Personality Inventory [MMPI]) of neuroticism, anxiety, rumination, depressed mood, introversion, poor coping skills, and dysfunctional attitudes and beliefs about sleep (Bonnet & Arand, 1997; Dorsey & Bootzin, 1997; Edinger, Fins, et al., 2000; Fernandez-Mendoza et al., 2011). In addition, the INSD phenotype is more likely to remit in its natural course (Vgontzas et al., 2012).

Developmentally, the INSD phenotype, defined in youth as having parent- or self-reported insomnia and sleeping objectively for >7.5 h, has been characterised in five studies by normal cortisol (Fernandez-Mendoza et al., 2014) and C-reactive protein (Fernandez-Mendoza et al., 2017) levels, yet increased beta EEG power during SOL (Fernandez-Mendoza et al., 2016a) and clinically elevated externalising behaviours (Calhoun et al., 2017; Fernandez-Mendoza, Calhoun, et al., 2016). In addition, the INSD phenotype in youth is less likely to worsen into adult insomnia disorder (Fernandez-Mendoza et al., 2022).

In terms of therapeutic response, the INSD phenotype showed better outcomes with CBT-I in the meta-analysis of seven retrospective RCTs mentioned above (He et al., 2022). Specifically, a 58% (95% CI 43%–73%) full remission rate that was two-times significantly higher than that of the ISSD phenotype reported above (He et al., 2022).

Finally, the identification of these two insomnia phenotypes has been supported by three data-driven cluster-analysis studies and one stability study. A study of PSG-measured TST, SOL and WASO in 100 individuals with insomnia disorder identified two clusters named INSD and ISSD (Miller et al., 2016). Within the ISSD cluster, investigators also identified an additional sub-cluster (ISSD-B), defined by high SOL and medium WASO, compared to the initially identified cluster (ISSD-A), which was defined by a high WASO (Miller et al., 2016). While both clusters had similar mean values on the Insomnia Severity Index (ISI), the ISSD phenotype had lower heart rate variability, greater reductions in delta, alpha, beta-1, and beta-2 EEG power at sleep onset (ISSD-B versus INSD), and worse performance on a task of sustained attention (ISSD-B versus ISSD-A and INSD). Subsequently, Miller and colleagues showed that the ISSD cluster had reduced concentrations of brain-derived metabolites, indicative of central hyperarousal, and poorer response to CBT-I than the INSD cluster (Miller et al., 2017; Miller et al., 2018). Another cluster-analysis study of PSG-measured TST, NREM spectral EEG power, and the interhemispheric asymmetry index in 99 individuals with insomnia disorder identified three clusters. The *first cluster* (26% of the sample) showed shorter TST and lower delta power and was named ‘short-sleep delta-deficient’ (SSDD), the *second cluster* (51%) showed normal TST and lower delta power and was named ‘normal-sleep delta-deficient’ (NSDD) and the *third cluster* (22%) showed normal TST and delta power and was named ‘normal neurophysiological sleep’ (NNS). In addition, acute sleep restriction improved subjective and PSG sleep across all three clusters, including increased sleep quality and delta power in SSDD and NSDD (Kao et al., 2021). The third cluster-analysis study examined ACT-measured sleep parameters in 103,000 subjects and identified three main cluster categories: long sleep duration, normal sleep duration, and short sleep duration (Katori et al., 2022), where the latter two clusters were further segmented into INSD and ISSD based on the presence of insomnia symptoms. As it pertains to phenotype stability, researchers have found that the relative classification of ISSD and INSD over the short-term (3 consecutive PSG nights) and long-term (2 PSG nights separated by 2.6 years) remains stable at a rate of 75% and 73%, respectively (Gaines et al., 2015).

#### 3.2.2 | Daytime sleep latency

The multiple sleep latency test (MSLT) is a measure of physiological sleep propensity where a shorter mean SOL (MSOL) from the four-to-five nap opportunities indicates greater physiological sleep propensity, with a threshold of ≤8 min recommended in the diagnosis of narcolepsy and idiopathic hypersomnia (ICSD-3). Individuals with insomnia disorder show a normal or even longer MSOL when compared to good sleepers (Bonnet & Arand, 1992; Bonnet & Arand, 1998b; Bonnet & Arand, 2000; Bonnet & Arand, 2005). In fact, the shorter the PSG-measured TST or lower sleep efficiency on the prior night, the longer the MSOL on the following day in individuals with insomnia disorder (Bonnet & Arand, 1998b; Roehrs et al., 2011). Roehrs and colleagues recently found that 87% of individuals with insomnia disorder with an MSOL of >11 min still had such a high MSOL 7 months later, suggesting that decreased physiological sleep propensity on the MSLT may be another valid measure of physiological hyperarousal in insomnia disorder (Bonnet & Arand, 1997; Bonnet & Arand, 2010; Roehrs et al., 2011). Based on such premise, four studies have used the MSLT to phenotype individuals with insomnia and investigate clinically relevant outcomes. Edinger et al. (2013) showed that individuals with insomnia disorder and a MSOL of >8 min, but not those with insomnia disorder and a MSOL of <8 min, made an average of 1.3 more errors on a complex reaction time task; a finding that investigators could not replicate in a subsequent study (Edinger et al., 2021). Li et al. (2015) showed that individuals with insomnia disorder and a MSOL of >14 min (OR = 3.27) or >17 min (OR = 4.33), but not those with insomnia disorder and a MSOL of <14 min (OR = 1.17), had increased odds of hypertension. Finally, Ren et al. (2021) showed that individuals with insomnia disorder and a MSOL of >14 min (OR = 1.89) or >17 min (OR = 3.73), but not those with insomnia disorder and a MSOL of <14 min (OR = 1.73), had increased odds of being underweight.

#### 3.2.3 | Daytime functioning

Multiple studies have investigated the association between insomnia and deficits in objective daytime functioning performance (Fortier-Brochu et al., 2012; Shekleton et al., 2010). However, no work has attempted to phenotype insomnia based on objective daytime performance (i.e., neurocognitive testing) using either a priori thresholds or cluster-analysis methods.

#### 3.2.4 | Other objective metrics

While several studies have investigated the association between insomnia and qEEG, only one has aimed at using qEEG alone, rather than in combination with PSG-measured TST (Kao et al., 2021), to identify phenotypes via data-driven cluster analysis. McCloskey et al. (2019) identified three clusters of insomnia distinguished by different peak frequencies from NREM stage 3 in the O1, F3, and C3 derivations; the *first cluster* was characterised by low beta EEG power, the *second cluster* by high delta EEG power, and the *third cluster* by low delta EEG power. However, these clusters were not linked to clinically relevant outcomes and no replication studies exist.

### 3.3 | Subjective measures in insomnia phenotyping

Given the recognised self-reported nature of insomnia as a diagnosis, subjective measures of night-time sleep, daytime functioning, and/or cognitive-emotional dimensions have also been a source of insomnia phenotyping efforts.

#### 3.3.1 | Insomnia symptoms

Insomnia has been historically classified based on the symptom type, such as sleep onset insomnia, sleep maintenance insomnia, and terminal/early morning insomnia. Among these symptoms, sleep maintenance insomnia has been reported as the most frequent, while terminal insomnia as the least common (Krystal, 2005; Manber & Ong, 2010). Classification based on these symptom types initially proved useful when selecting specific pharmacological agents; however, because many of the insomnia symptoms tend to cluster together (Krystal, 2005) and do not remain stable over time (Pillai et al., 2015), this method of insomnia phenotyping did not provide an adequate basis for classification. Consequently, none of the DSM and ICSD nosologies used symptom types to phenotype insomnia, while they remain integral to the diagnosis of the disorder (Table 1).

Insomnia has also been classified based on how long individuals report having experienced symptoms, providing initial phenotypes of transient (<2 weeks), short-term (2–4 weeks) and chronic (>4 weeks) insomnia (Krystal, 2005), as well as the introduction of adjustment sleep disorder and idiopathic insomnia in the ICSD-R. The current ICSD-3 and DSM-5-TR include duration-based phenotypes of chronic/persistent and acute/short-term insomnia disorder (Table 2). While several studies have provided support for the distinction between these duration-based phenotypes (Ellis et al., 2012a; Ellis et al., 2012b), the duration itself has provided little guidance to clinicians in determining whether interventions should be distinct (behavioural versus pharmacological) based on symptom duration or acute versus chronic nature of insomnia, given that acute insomnia is part of the natural history of chronic insomnia disorder and may also require CBT-I as first-line treatment (Buysse, 2010; Ellis, 2019; Ellis et al., 2015).

#### 3.3.2 | Night-time sleep duration

A total of 22 studies had examined the adverse health outcomes in individuals with the proposed ISSD and INSD phenotypes using subjective TST to define them (Ballesio et al., 2019; Bansil et al., 2011; Canivet et al., 2014; Cespedes et al., 2016; Chandola et al., 2010; Clark et al., 2014; Daghlas et al., 2019; Furihata et al., 2020; Helbig et al., 2015; Hoevenaar-Blom et al., 2011; Kalmbach et al., 2016; Lou et al., 2012; Lou et al., 2014; Lou et al., 2015; Lu et al., 2015; Makino et al., 2018; Sands-Lincoln et al., 2013; Shekleton et al., 2014; Sivertsen et al., 2014; Vozoris, 2013; Westerlund et al., 2013). Although these data may be consistent with those based on objective TST in some studies with large sample sizes, studies that have concomitantly used objective and subjective measures of TST in the same sample of individuals with insomnia have shown that subjective TST shows lower accuracy, poorer precision, and poorer discriminant value than objective TST when predicting pathophysiological (Jarrin et al., 2018b), cardiovascular (Bathgate et al., 2016; Bertisch et al., 2018), and therapeutic (Bathgate et al., 2017) outcomes for the ISSD phenotype, which can be explained by the differential distribution of sleep misperception across the INSD and ISSD phenotypes reviewed above (e.g., Fernandez-Mendoza et al., 2011).

#### 3.3.3 | Daytime functioning

The type and severity of daytime symptoms reported by individuals with insomnia is also highly heterogenous; thus, distinctive daytime symptom profiles may identify insomnia phenotypes. Using profile analysis via multidimensional scaling in a sample of 332 individuals with insomnia disorder, Sánchez-Ortuño et al. (2011) identified four prototypical patterns of self-reported daytime symptoms:

- The *first prototype* was characterised by poor daytime sleep hygiene behaviours, high tension/anxiety, and low fatigue.
- The *second prototype* was the mirror image of the *first prototype*.
- The *third prototype* was characterised by mood disturbance and low sleepiness.
- The *fourth prototype* was the mirror image of the *third prototype*.

These four prototypes distributed differently across the ICSD-2 insomnia phenotypes. While psychophysiological insomnia and inadequate sleep hygiene were associated with the *first prototype*, idiopathic insomnia and insomnia related to a mental disorder were associated with the *second prototype*. Paradoxical insomnia was not significantly associated with any specific daytime profile, but rather with less severe overall self-reported daytime symptoms. These data led Sánchez-Ortuño et al. (2011) to hypothesise the presence of two insomnia phenotypes, each with a distinct underlying pathophysiology: the similar daytime prototype shared by idiopathic insomnia and insomnia associated to a mental disorder could be indicative of a form of insomnia arising from an endogenous factor (i.e., a ‘neurophysiological or neurochemical’ insomnia), while the similar daytime prototype shared by psychophysiological insomnia and inadequate sleep hygiene could be indicative of another form of insomnia arising from maladaptive sleep behaviours (i.e., a ‘learned’ insomnia). No hypothesised pathophysiology was provided for the overall milder daytime symptoms prototype exhibited by paradoxical insomnia, other than its potential congruency with the INSD phenotype reviewed above. However, these prototypes were not linked to clinically relevant health or treatment outcomes and no replication studies exist.

#### 3.3.4 | Cognitive-emotional traits and life history

Historically, insomnia has been viewed primarily as a biobehavioural disorder (Kales et al., 1976; Kales et al., 1983; Spielman et al., 1987). With this view, insomnia develops when acute emotional stress (precipitating factors) produces poor sleep in vulnerable individuals (predisposing factors) and becomes persistent when inappropriate behavioural responses to the poor sleep are learned (perpetuating factors) (Spielman et al., 1987). Seminal studies showed characteristic cognitive-emotional or personality traits in patients with insomnia. Assessments of psychological profile patterns or code types using the MMPI in patients with insomnia revealed different but consistent personality patterns (Kales et al., 1976; Kales et al., 1983; Marks & Seeman, 1963). Kales and colleagues identified six frequent MMPI code-types in people with insomnia that characterised them as ‘individuals who internalised emotion, were unable to discharge anger outwardly, dysthymic, apprehensive, inhibited, and ruminative’. These findings were similar when relying on other personality tests (Dorsey & Bootzin, 1997); however, these prior efforts relied on MMPI profiling using codes, rather than data-driven statistical tools.

Early cluster analysis work utilised MMPI clinical scales and identified two clusters among 100 individuals with insomnia (Edinger et al., 1988):

- The *first cluster* consisted of individuals with insomnia that were more willing to admit personal faults and shortcomings, less psychologically defended, reported fewer somatic concerns, were more activated/aroused, more prone to report childhood sleep disturbances, and to present for treatment in their late 30’s. Primary symptoms for these patients included sleep-disruptive cognitions, long periods of wakefulness in bed, agonising about sleep, and being resistant to behavioural treatments for their sleep problems.
- The *second cluster* consisted of individuals with insomnia who had clearly defined ‘neurotic’ profiles with high somatic concerns, depression, hysteria, with the onset of sleep difficulties occurring during adulthood, who did not present for treatment until their early 50’s, and in whom behavioural treatments were effective in addressing their sleep difficulties.

Another study conducted data-driven cluster analysis of the 16-item Dysfunctional Beliefs and Attitudes About Sleep Scale and compared treatment outcomes among the four identified clusters in 104 normal sleeping and 281 insomnia subjects meeting the DSM-IV-TR criteria for insomnia (Sánchez-Ortuño & Edinger, 2010):

- The *first cluster* comprised individuals with an above-normal range for worry/helplessness, negative consequences, and sleep medication beliefs and was named ‘worried and medication-based’.
- The *second cluster* comprised individuals less likely to endorse the negative consequence of insomnia belief and was named ‘low endorsement’.
- The *third cluster* comprised individuals who were within an abnormal range on the worry/helplessness beliefs and was named ‘mild sleep worries’.
- The *fourth cluster* comprised individuals who had strong beliefs on the negative consequences and worry/helplessness beliefs and was named ‘worried and symptom-focused’. After 6-months of follow-up, individuals within the identified clusters had responded differently to CBT-I, with this *fourth cluster* exhibiting a better treatment response, including remission as measured by the Insomnia Symptom Questionnaire, compared to the other clusters.

Using broader and more extensive subjective measures of personality (NEO Five-Factor and Temperament and Character Inventories), sleep (sleep diary and ISI), psychopathology (semi-structured interview), and fatigue (Checklist of Individual Strengths), van de Laar et al. (2017) identified three clusters in 218 individuals:

- The *first cluster* self-reported moderate ISI scores and normal scores on personality inventories, normal anxiety, and depressive symptoms.
- The *second cluster* self-reported severe ISI scores and a 20% lower subjective sleep efficiency, 12–14 h less subjective TST per week, and two-times higher subjective SOL compared to other groups, and slightly higher anxiety and depressive symptoms, higher scores on harm avoidance, below average scores on self-directedness, low self-sufficiency, and were less goal-directed than the moderate insomnia group.
- The *third cluster* self-reported an earlier age of onset for their insomnia (16 years younger at onset of insomnia), were ~10 years younger and had insomnia for longer than individuals with the other phenotypes; additionally, they were more likely to self-report higher anxiety and depression, higher neuroticism and lower extroversion and conscientiousness personality scores, and higher harm avoidance and lower self-directedness temperament and character scores.

**FIGURE 3:**
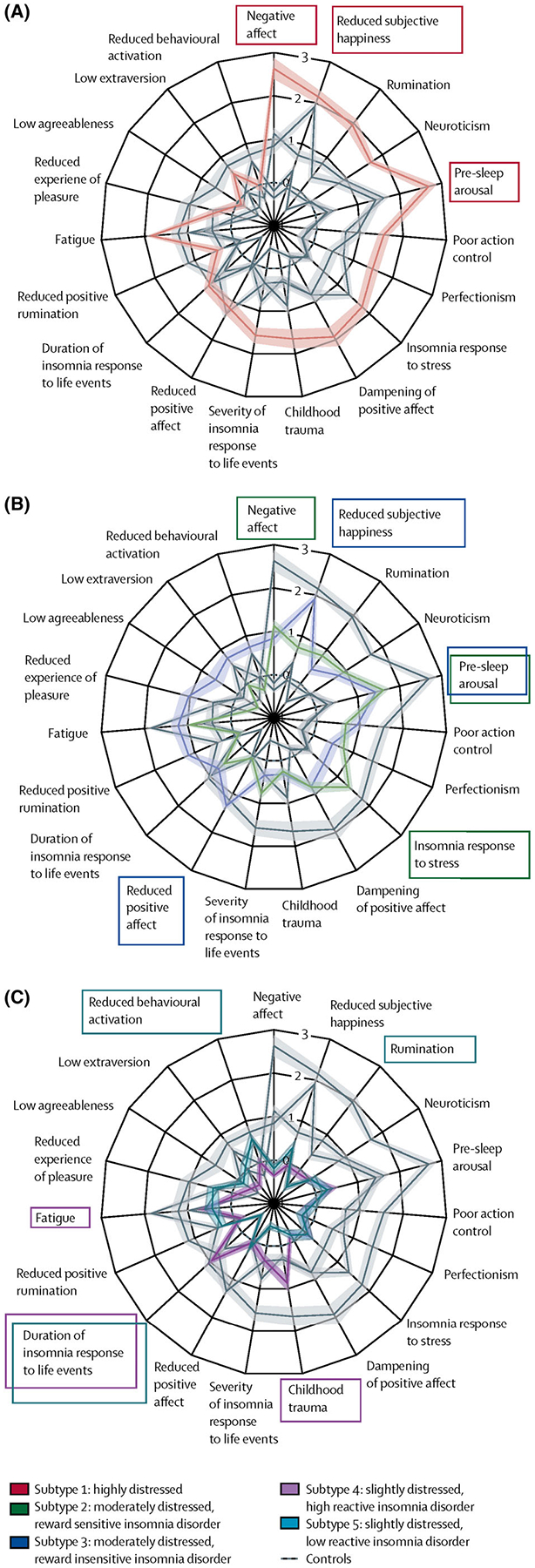
Insomnia phenotypes based on cognitive-emotional traits and life history. Data are scaled phenotype group means (95% confidence intervals), in which *Z* scores have been standardised to the mean and standard deviation of controls for each characteristic, with the phenotype-explained variance, ranked clockwise from the top. (a) Highly distressed phenotype (phenotype 1). (b) Moderately distressed phenotypes (phenotype 2, which was reward sensitive, and phenotype 3, which was reward insensitive). (c) Low distress phenotypes (phenotype 4, which was high reactive, and phenotype 5, which was low reactive). Positive characteristics (e.g., positive rumination) were reverse-coded and renamed (e.g., reduced positive rumination), such that higher values uniformly indicate higher general distress for all characteristics throughout the plot. Coloured boxes indicate the three characteristics that differentiate each phenotype the most from control participants. Taken with permission from Blanken TF et al. (2019). *Insomnia disorder subtypes derived from life history and traits of affect and personality*. The Lancet Psychiatry, 6(2), 151–163.

As shown in Figure 3, more recent work from Blanken et al. (2019) identified five insomnia clusters by examining online survey data on sleep, personality, life events, and health conditions from 34 existing validated questionnaires completed by 1,046 participants aged ≥18 years. These survey data were consolidated to develop and validate, in a non-overlapping cohort, the Insomnia Type Questionnaire (ITQ):

- The *first cluster*, termed ‘highly distressed’, included individuals with high general distress, high pre-sleep arousal, negative affect, and reduced subjective happiness, among multiple other features.
- The *second cluster*, termed ‘moderately distressed and reward sensitive’, included individuals with high pre-sleep arousal, stress-related sleep reactivity, and negative affect.
- The *third cluster*, termed ‘moderately distressed and reward insensitive’, comprised of individuals who also had high pre-sleep arousal, but had significantly reduced subjective happiness and positive affect compared to the *second cluster*.
- The *fourth cluster*, termed ‘slightly distressed and highly reactive’, was comprised of individuals who had lower distress levels compared to other clusters except more frequent childhood trauma and a longer duration of insomnia as a response to life events.
- The *fifth cluster*, termed ‘slightly distressed and low reactive’, comprised of those individuals who also had lower distress levels and longer duration of insomnia as a response to life events, but also had significantly lower behavioural activation and rumination compared to the *fourth cluster*.

There were also differences between the five clusters with respect to the severity of insomnia symptoms. Half of those in the *first* and *second clusters* reported difficulty initiating sleep in their teenage years, whereas half of the individuals in the *fourth* and *fifth clusters* reported difficulty initiating sleep by their 40’s. In addition, individuals in the *fifth cluster* experienced diseases of the CNS more frequently than the other clusters, with the *second cluster* experiencing fewer CNS diseases compared to other clusters. With respect to self-reported efficacy of hypnotic medication, individuals in the *second* and f*ourth clusters* experiencing difficulty maintaining sleep were more likely to report improvement after benzodiazepine intake. However, only those individuals in the *second cluster* reported an improvement in their ability to initiate sleep; no improvement in difficulty initiating sleep was reported by individuals within the fourth-phenotype group associated with hypnotic medication intake. Lastly, when using an auditory oddball paradigm, individuals in the *fourth cluster* demonstrated evidence of hyperactive late processing compared to controls.

## 4 | DISCUSSION

This systematic review shows that current nosology has adopted a single diagnosis approach for insomnia, despite the long-term recognition that it is a heterogeneous disorder. The different phenotypes delineated by the DSM and ICSD from 1987 until 2014 did not show optimal diagnostic reliability and validity (Edinger et al., 2011) or any accompanying clinically relevant outcomes (Buysse et al., 1998). Historically, the ICSD-R and ICSD-2, in their ‘splitting’ approach to insomnia nosology, suggested using PSG to aid in the diagnosis of some of its phenotypes in adults; namely, psychophysiological, paradoxical, and idiopathic insomnia. However, the lack of specific PSG thresholds within the ICSD and ICSD-R to define these phenotypes, the changes made in their criteria across different iterations of the ICSD-R and ICSD-2 or in practice guidelines not recommending PSG for the clinical assessment of insomnia (Schutte-Rodin et al., 2008), all likely contributed to the low diagnostic reliability and validity of those ICSD phenotypes over time (Edinger et al., 2011). In addition, this review shows that while self-reported insomnia symptoms of difficulty initiating sleep, difficulty maintaining sleep, and early morning awakening remain as an integral feature of the diagnosis of insomnia disorder in the DSM-5-TR and ICSD-3, there is currently no evidence to suggest that the type of self-reported symptom pattern is a clinically meaningful way of phenotyping the disorder, beyond tailoring short- and long-acting sleep medications (Krystal, 2005). More recently, objective measures of sleep, including PSG, have been proposed for identifying insomnia phenotypes with distinct biological severity (Fernandez-Mendoza, 2017; Fernandez-Mendoza, 2022; Fernandez-Mendoza & Vgontzas, 2013; Vgontzas et al., 2013). In addition, there has been a renewed interest in the past few years in applying cluster-analytic techniques to identify insomnia phenotypes based on objective (Kao et al., 2021; Katori et al., 2022; McCloskey et al., 2019; Miller et al., 2016) or subjective (Blanken et al., 2019; Edinger et al., 1988; Sánchez-Ortuño & Edinger, 2010; van de Laar et al., 2017) night-time sleep, daytime functioning, and/or cognitive-emotional data. Overall, the use of objective measures of night-time sleep duration and the use of subjective measures of cognitive-emotional traits have been identified within this systematic review as the two most promising areas of insomnia phenotyping and will be critically appraised below.

The current use of objective sleep measures has enabled the identification of the INSD and ISSD phenotypes, which were derived using a multidimensional (insomnia symptoms, sleep duration) and multimethod (subjective, objective) approach that has attributed distinct pathophysiology, risk of specific types of morbidity, clinical features, natural course, and treatment response to each phenotype. As shown in this review, support for this insomnia phenotyping comes from 66 studies covering each of those clinically relevant domains; 44 studies have supported the association of ISSD with physiological hyperarousal, with increased cardiometabolic and neurocognitive risk and a persistent, long-lasting natural course. A meta-analysis of seven RCTs supports a blunted response to CBT-I in the ISSD phenotype; however, these data were all retrospective secondary analyses and there is no evidence from prospective RCTs designed to test the differential response of the ISSD and INSD phenotypes to CBT-I or other insomnia therapies. A novel aspect in this literature is that four studies using latent class approaches have provided support for the replicability of the ISSD and INSD phenotypes by independent researchers and across different populations. Unsupervised, unbiased cluster-analysis studies are important because they implement a data-driven approach and do not use a priori thresholds (e.g., <6 h of TST). Overall, these studies identified the ISSD and INSD phenotypes via PSG (Miller et al., 2016) and PSG *plus* qEEG (Kao et al., 2021) in the same clinical cohort and via ACT in the UK Biobank (Katori et al., 2022). Interestingly, while Miller et al. (2016) identified two clusters of the ISSD phenotype, named ISSD-A and ISSD-B, distinguished by their level of PSG-measured WASO and SOL, Kao et al. (2021) identified the ISSD phenotype, which was delta power deficient based on NREM qEEG (named SSDD), and two clusters of the INSD phenotype, named NSDD and NNS, and distinguished by their level of NREM qEEG-measured delta power (delta deficient versus not). This additional work suggests that including more objective measures may not result in replicable subgroups of the ISSD and INSD phenotypes within the same cohort; thus, more reliable, and replicable work needs to be done if investigators aim to sub-phenotype ISSD and INSD, which appears premature at this juncture. The extant literature, including replication studies and meta-analyses, supports clinically meaningful differences between the ISSD and INSD phenotypes when defined based on PSG-measured TST; however, PSG is burdensome, inconvenient, and costly, even when performed at home. Several researchers have investigated potential replacements or supplements to night-time PSG-measured TST by using MSLT-measured daytime sleep propensity, subjectively-measured TST or ACT-measured TST. Insomnia phenotyping based on the MSLT is also a multidimensional (insomnia symptoms, alertness) and multimethod (subjective, objective) approach, with support for this insomnia phenotyping coming from three studies covering clinically relevant domains. However, there are no replication studies for cardiometabolic outcomes or data-driven cluster-analysis studies of the MSLT to phenotype insomnia. Additionally, implementing the MSLT already requires a PSG on the prior night, being costly and clinically impractical despite its demonstrated scientific value. Thus, more reliable, and replicable measures of 24-h physiological hyperarousal should be tested and validated for insomnia phenotyping. Subjective night-time TST has also been investigated, likely due to it being a more cost-effective alternative to objective measurement; however, extant evidence shows that subjective TST does not reliably capture the degree of physiological sleep ability (i.e., short objective TST) that distinguishes the ISSD and INSD phenotypes. This is due, in part, to the fact that individuals with INSD and ISSD both report a similar amount of subjective TST and significant underestimation of sleep duration (i.e., so-called sleep misperception) is a clinical feature of those with INSD (Fernandez-Mendoza et al., 2011; Jarrin et al., 2018a; Krystal et al., 2002). These issues could have contributed to the mixed and contrasting findings seen in studies examining subjective sleep duration and CBT-I treatment response (Galbiati et al., 2018) or adherence (Ong et al., 2008; Perlis et al., 2000; Yeung et al., 2015). As reviewed above, several studies have defined the ISSD and INSD phenotypes using ACT-measured TST (Bathgate et al., 2017; Castro-Diehl et al., 2015; Castro-Diehl et al., 2016; Castro-Diehl et al., 2018; Floam et al., 2015; Katori et al., 2022; Lerman et al., 2022; Nakazaki et al., 2012; Vgontzas et al., 2020). Evidence supports the use of the same threshold as for PSG to define the ISSD phenotype (i.e., <6 h in adults); however, ACT is known to significantly overestimate PSG-measured TST, so it is likely that a threshold of <6.5 or 7 h in adults may be needed to reliably identify the ISSD phenotype (Fernandez-Mendoza, 2022). Thus, ACT and similar ambulatory devices with expanded physiological signals, including less burdersome PSG, are well-positioned to change nosology and introduce their use in phenotyping ISSD and INSD; however, this is an area that deserves systematic reliability studies.

The improvements made in latent class analyses over the past decades has enabled the identification of clusters of insomnia that may index phenotypes, an approach used in four studies using objective sleep measures, as reviewed above, and in four studies using subjective sleep and daytime measures. Phenotyping of insomnia based on data-driven cluster analysis of cognitive-emotional traits is a multidimensional approach that has attributed specific clinical features to the resulting phenotypes. More specifically, van de Laar et al. (2017) reported that earlier age of insomnia onset was associated with higher psychiatric comorbidity and Blanken et al. (2019) provided evidence that two of the five clusters differed in their self-reported benefit from using hypnotic medication for their sleep difficulties. However, these cognitive-emotional cluster-analysis studies have not complemented their multidimensional approach with a multimethod measurement, like other multidimensional sleep health approaches are moving towards (Hall et al., 2022). Also, these studies have not linked each cluster (phenotype) to other clinically relevant outcomes not measured by patient-reported outcomes, such as pathophysiological biomarkers, physical health, neurocognitive impairment, or response to CBT-I. In addition, all four cluster-analysis studies used different cognitive-emotional domains and self-reported measures, making it difficult to determine if they were able to identify similar and equivalent phenotypes across them. Consequently, there are no replication studies for each identified cluster across studies or prospective examination of the impact of these clusters on health outcomes or treatment response. Importantly, Blanken et al. (2019) were not only able to identify five insomnia clusters, but they also developed a corresponding ITQ, which they employed to test the stability of the identified clusters over time. Investigators showed that their clusters remained stable after an average of 4.8 years, with 87% stability in cluster classification. However, many of the self-reported features reported by Blanken and colleagues in the identified clusters (e.g. recurrent nightmares, confusional arousals, higher prevalence of psychopathology, or childhood-onset personality disorders), may not be ‘phenotypic’ of insomnia, but rather comorbidities with their own aetiology and pathophysiology not otherwise excluded from the analyses (e.g., comorbid trauma-related nightmare disorder). Finally, the ITQ is comprised of 200 items, which may be perceived as burdensome by study participants, researchers, patients, and clinicians alike. Thus, more reliable, and replicable work needs to be done across different populations using the ITQ or similar multidimensional packets of cognitive-emotional measures, given that cluster analysis provides as rich information as the measures that are included or excluded in the data collection.

As a final note, we have used in the present systematic review the terms ‘phenotypes’ and ‘phenotyping’ as synonymous to ‘subtypes’ and ‘subtyping’ because the insomnia literature has used them interchangeably. Some authors have used terms such as ‘types’ to refer to the insomnia symptoms patterns or the nosology categories reviewed herein (Perlis et al., 2022). Nevertheless, phenotyping typically refers to defined groups within a disorder with distinct pathophysiology and clinical features, while subtyping typically refers to defined groups with quantitatively greater or lower degree of essential or associated symptoms within a disorder.

In conclusion, having adequate diagnostic systems to properly identify individuals with specific phenotypes enables properly tracking prevalence, persistence, and incidence rates, investigate their pathophysiology, assess their independent contribution to other adverse health outcomes, and test alternative and improved treatment options. Nosology should guide treatment to increase success, measured by improved signs and symptoms as well as patient’s quality of life. The current guideline-recommended first-line treatment for insomnia, CBT-I, is efficacious in RCTs, effective long-term, can be delivered without face-to-face interaction with therapists, and is poised to widespread dissemination across disciplines and specialties and clinical and community settings. Yet, it leaves ~50% of individuals with insomnia disorder unremitted. Current clinical guidelines recommend a course of action where patients undergo CBT-I and, if unsuccessful, pharmacotherapy should be introduced. An improved phenotyping of insomnia could change this course of action by either matching therapy modalities to each phenotype, improving existing therapies to match phenotype-specific needs, or combining therapy modalities (pharmacotherapy and psychotherapy) for specific phenotypes. Any of these options should be performed a priori, not once patients have failed a given therapy. This systematic review shows a worldwide effort, and significant advances, in this direction.

## Figures and Tables

**TABLE 1:** Evolution of general insomnia diagnostic criteria in prior and current nosology

Criterion	DSM-III-R (1987)	ICSD-R (1997)	DSM-IV-TR (2000)	RDC (2004)	ICSD-2 (2005)	ICSD-3 (2014)	DSM-5-TR (2022)
Criterion A	Difficulty initiating or maintaining sleep, or of nonrestorative sleep	None	None	Complaints associated with one or more of the following: difficulty initiating sleep, difficulty maintaining sleep, waking too early, or chronically nonrestorative or poor quality	Complaint of difficulty initiating sleep, maintaining sleep, or waking too early, or sleep that is chronically non-restorative or in poor quality. In children, reported by caretaker and consist of observed bedtime resistance or inability to sleep independently.	Complaints associated with one or more of the following: difficulty initiating sleep, maintaining sleep (in children, difficulties initiating or maintaining sleep may include resistance to going to bed on appropriate schedule and/or difficulty sleeping without parent or caregiver), waking up too early	Predominant complaint of dissatisfaction with sleep quantity or quality, associated with one or more of the following symptoms: difficulty initiating sleep, difficulty maintaining sleep (in children difficulties initiating or maintaining sleep may manifest without caregiver intervention), or early-morning awakening with inability to return to sleep
Criterion B	Sleep difficulty occurs ≥3 times/week for ≥1 month and sufficiently severe to result in either a complaint of daytime fatigue or the observation by others of some symptom that is attributable to the sleep disturbance, e.g., irritability or impaired daytime functioning	None	None	Complaint occurs despite adequate opportunity or circumstance for sleep	Sleep difficulty occurs despite adequate opportunity and circumstance for sleep	Disturbance causes at least one form of daytime impairment related to night-time sleep difficulty	Disturbance causes clinically significant distress or impairments in social, occupational, educational, academic, behavioural, or other important areas
Criterion C	Occurrence not exclusively during the course of sleep-wake schedule disorder or a parasomnia	None	None	Disturbance causes at least one form of daytime impairment related to night-time sleep difficulty	At least one form, out of nine, of daytime impairment related to the night-time sleep difficulty is reported by patient.	Complaints cannot be purely explained by inadequate opportunity or circumstances for sleep	Difficulty occurs ≥3 times/week
Criterion D	None	None	None	None	Difficulty present for ≥ 1 month	Disturbance and associated daytime symptoms occur ≥3 times/week	Difficulty present for ≥3 months
Criterion E	None	None	None	None	None	Disturbance and associated daytime symptoms have been present for ≥3 months	Difficulty occurs despite adequate sleep opportunity
Criterion F	None	None	None	None	None	Difficulties not better explained by another sleep disorder	Not better explained or does not occur in course of another sleep-wake disorder
Criterion G	None	None	None	None	None	None	Not attributable to physiological effects of substance
Criterion H	None	None	None	None	None	None	Co-existing mental disorders and medical conditions do not adequately explain predominant insomnia complaint

Abbreviations: DSM-III-R, Diagnostic and Statistical Manual of Mental Disorders, 3rd Edition, Revised; DSM-IV-R, Diagnostic and Statistical Manual of Mental Disorders, Fourth Edition, Revised; DSM-5-TR, Diagnostic and Statistical Manual of Mental Disorders, Fifth Edition, Revised; ICSD-2, International Classification of Sleep Disorders, Second Edition; ICSD-3, ICSD-R, International Classification of Sleep Disorders, Third Edition; International Classification of Sleep Disorders, Revised; RDC, Research Diagnostic Criteria.

**TABLE 2:** Evolution of specific insomnia diagnostic phenotypes in prior and current nosology

	DSM-III-R (1987)	ICSD-R (1997)	DSM-IV-TR (2000)	RDC (2004)	ICSD-2 (2005)	ICSD-3 (2014)	DSM-5-TR (2022)
Primary versus secondary	Primary insomnia Insomnia related to another mental disorder (non-organic) Insomnia related to a known organic factor	Environmental sleep disorder Altitude insomnia Food allergy Insomnia Sleep disorder associated with mental disorders Sleep disorder associated with neurological disorders Sleep disorder associated with other medical disorders	Primary insomnia Insomnia related to another mental disorder Sleep disorder due to a general medical condition Insomnia type Substance-induced sleep disorder Insomnia type Insomnia due to another sleep disorder	Primary insomnia Insomnia due to a mental disorder Insomnia due to medical condition Insomnia related to periodic limb movement disorder Insomnia related to sleep apnea Insomnia due to drug or substance use	Insomnia due to a mental disorder Insomnia due to medical condition Insomnia due to drug or substance	None	None
Sleep hygiene	None	Inadequate sleep hygiene	None	None	Inadequate Sleep Hygiene	None	None
Conditioning/objective sleep	None	Psychophysiological insomnia	None	Psychophysiological Insomnia	Psychophysiological Insomnia	None	None
Sleep discrepancy	None	Sleep state misperception	None	Paradoxical Insomnia	Paradoxical Insomnia	None	None
Age of onset	None	Idiopathic insomnia Limit-setting sleep disorder Sleep-onset association sleep disorder	None	Idiopathic insomnia	Idiopathic insomnia Behavioural insomnia of childhood	None	None
Chronicity	None	Adjustment sleep disorder		None	Adjustment insomnia (acute insomnia)	Chronic insomnia disorder Acute insomnia disorder	Insomnia disorder, episodic Insomnia disorder, persistent Insomnia disorder, recurrent Other specified insomnia disorder (acute and short-term)

Abbreviations: DSM-III-R, Diagnostic and Statistical Manual of Mental Disorders, 3rd Edition, Revised; DSM-IV-R, Diagnostic and Statistical Manual of Mental Disorders, Fourth Edition, Revised; DSM-5-TR, Diagnostic and Statistical Manual of Mental Disorders, Fifth Edition, Revised; ICSD-2, International Classification of Sleep Disorders, Second Edition; ICSD-3, ICSD-R, International Classification of Sleep Disorders, Third Edition; International Classification of Sleep Disorders, Revised; RDC, Research Diagnostic Criteria.

**TABLE 3:** Summary of sleep microstructure studies on psychophysiological insomnia (Psy-I) and paradoxical insomnia (Para-I) as per International Classification of Sleep Disorders, Second Edition diagnostic criteria

	Para-I (*n* ~ 30)	Psy-I (*n* ~ 30)	Para-I ≠ controls	Psy-I ≠ controls	Para-I ≠ Psy-I
Insomnia Severity Index, score	17.6	16.6	Yes	Yes	No
Diary-measured SOL, min	46.0*	24.0	Yes	Yes	Yes
Diary-measured WASO, min	126.5*	60.3	Yes	Yes	Yes
Diary-measured TST, h	315.8*	401.2	Yes	Yes	Yes
Diary-measured SE, %	65.1*	82.8	Yes	Yes	Yes
PSG-measured SOL, min	11.7	10.5	No	No	No
PSG-measured WASO, min	52.8	56.3	Yes	Yes	No
PSG-measured TST, h	411.0	401.9	No	No	No
PSG-measured SE, %	86.0	85.0	Yes	Yes	No
Arousal index, per h	31.7	18.3	Yes	Yes	Yes
CAP-total rate, %	58.1	49.6	Yes	Yes	Yes
Delta, μV^2^	2.4*	2.3	Yes	Yes	Yes
Theta, μV^2^	1.7	1.6	No	No	No
Alpha, μV^2^	1.5*	1.4	Yes	Yes	Yes
Sigma, μV^2^	−0.5	−0.6	Yes	No	No
Beta, μV^2^	0.5*	0.4	Yes	Yes	Yes
Sleep spindles duration, ms	0.6	0.7	Yes	No	No
N1 evoked potential in REM	−2.2*	−1.1	No	No	Yes
P2 evoked potential in REM	4.2*	2.5	Yes	No	Yes
Wake intrusions in REM, *n*	2.3	3.6*	No	No	Yes

*Note*: Data comes from C. Bastien’s studies (Bastien et al., 2003; Bastien et al., 2008; Bastien et al., 2009b; Bastien et al., 2009a; Bastien et al., 2013), except cyclic alternating pattern from L. Parrino (Parrino et al., 2009), I. Chouvarda (Chouvarda et al., 2013), and A. Rodenbeck (Rodenbeck et al., 2000). C. Bastien reviewed an initial version of this summary table in May 2018.

Abbreviations: CAP, cyclic alternating pattern; PSG, polysomnography; REM, rapid eye movement; SE, Sleep efficiency; SOL, Sleep onset latency; TST, Total sleep time; WASO, wake after sleep onset.

## Data Availability

Data will be made available upon reasonable request to the corresponding author.
